# Anti-Photoaging Effect of Hydrolysates from Pacific Whiting Skin via MAPK/AP-1, NF-κB, TGF-β/Smad, and Nrf-2/HO-1 Signaling Pathway in UVB-Induced Human Dermal Fibroblasts

**DOI:** 10.3390/md20050308

**Published:** 2022-04-30

**Authors:** Seok Hee Han, Elaine Ballinger, Se-Young Choung, Jung Yeon Kwon

**Affiliations:** 1 Department of Biomedical and Pharmaceutical Sciences, Graduate School, Kyung Hee University, 26 Kyungheedae-ro, Dongdaemun-gu, Seoul 02447, Korea; idcass@khu.ac.kr (S.H.H.); sychoung@khu.ac.kr (S.-Y.C.); 2 Department of Food Science and Technology, Oregon State University, Corvallis, OR 97331, USA; elaine.ballinger@oregonstate.edu; 3 Seafood Research and Education Center, Oregon State University, Astoria, OR 97103, USA; 4 Department of Preventive Pharmacy and Toxicology, College of Pharmacy, Kyung Hee University, 26 Kyungheedae-ro, Dongdaemun-gu, Seoul 02447, Korea

**Keywords:** *Merluccius productus*, Matrix metalloproteinase, type I procollagen, MAPK/AP-1, NF-κB, Nrf-2/HO-1, TGF-β/Smad

## Abstract

Chronic exposure to ultraviolet (UV) light promotes the breakdown of collagen in the skin and disrupts the extracellular matrix (ECM) structure, leading to skin wrinkling. Pacific whiting (*Merluccius productus*) is a fish abundant on the Pacific coast. In the current study, we investigated the anti-wrinkle effect of hydrolysate from Pacific whiting skin gelatin (PWG) in UVB-irradiated human dermal fibroblasts and the molecular mechanisms involved. PWG effectively restored type 1 procollagen synthesis reduced by UVB-irradiation. Also, we found that PWG inhibited collagen degradation by inhibiting MMP1 expression. Furthermore, PWG decreased cytokines TNF-α, IL-6, and IL-1β associated with inflammatory responses and increased antioxidant enzymes, HO-1, SOD, GPx, CAT, and GSH content, a defense system against oxidative stress. In terms of molecular mechanisms, PWG increased collagen synthesis through activating the transforming growth factor β (TGF-β)/Smad pathway and decreased collagen degradation through inhibiting the mitogen-activated protein kinases/activator protein 1 (MAPK/AP-1) pathway. It also suppressed the inflammatory response through suppressing the nuclear factor-κB (NF-κB) pathway and increased antioxidant enzyme activity through activating the nuclear factor erythroid 2/heme oxygenase 1 (Nrf-2/HO-1) pathway. These multi-target mechanisms suggest that PWG may serve as an effective anti-photoaging material.

## 1. Introduction

Skin aging is divided into intrinsic aging and extrinsic aging [1]. Intrinsic aging is highly correlated with genetic factors [2]. Conversely, extrinsic aging is caused by several environmental factors, such as ultraviolet (UV), air pollution, chemicals, and environmental toxins. Among them, the biggest contributing factor of skin aging is UV exposure. Ultraviolet consists of wavelengths from 200 to 400 nm and is divided into three parts: UVA (320–400 nm), UVB (280–320 nm), and UVC (200–280 nm). UVA and UVB have biological effects on the skin, and while UVA occupies most of the UV rays, it is weakly carcinogenic. UVB penetrates deeply into the epidermis and dermis and is more biologically dangerous [3]. Repeated exposure to UVB affects the fibroblasts present in the dermis. Fibroblasts are involved in collagen synthesis and occupy most of the connective tissue [4]. Therefore, photodamaged skin characterizes thick and deep wrinkles, skin dryness, and loss of elasticity [5]. This accelerated aging of the skin due to the accumulation of continuous UV exposure is termed skin photoaging [2].

UV irradiation increases the skin’s matrix metalloproteinases (MMPs) to break down ECM proteins. MMPs are a large family of calcium-dependent zinc-containing endopeptidases responsible for the degradation and remodeling of the ECM [6]. Among them, MMP1 specifically degrades type 1 collagen. It is regulated by MAPKs, an upstream signaling cascade consisting of extracellular signal regulated kinase (ERK), c-Jun N-terminal kinase (JNK), and p38. MAPKs activated by UV irradiation increase the expression of AP-1, a transcription factor of MMPs composed of Jun and Fos heterodimers. Another important MMP transcription factor, nuclear factor kappa B (NF-kB), is localized to the cytoplasm by IκBα, but photodamaged by UVB irradiation, resulting in dissociation of IκBα and translocation of NF-kB to the nucleus. Translocated NF-κB increases pro-inflammatory cytokines such as tumor necrosis factor-α (TNF-α), interleukin-6 (IL-6), and interleukin-1β (IL-1β) contribute to skin damage [7]. UVB also affects the TGF-β/Smad pathway, a representative collagen synthesis pathway. Transforming growth factor-β (TGF-β) stimulates the biosynthesis of type 1 collagen and regulates collagen homeostasis through Smad signaling molecules [8,9]. Reactive oxygen species (ROS) produced by UV irradiation damages the TGF-β signaling pathway by reducing transforming growth factor-β receptor II (TGF-βRII) expression and downregulating Smad3 phosphorylation [10]. UVB-induced TGF-β/Smad pathway damage is one of the mechanisms causing the loss of collagen. The increase in ROS due to continuous UV irradiation is accompanied by oxidative stress [11]. Nuclear factor erythroid 2-related factor 2 (Nrf-2) can scavenge ROS and is an antioxidant protection system against oxidative stress [12]. In the normal state, Nrf-2 forms a complex with Keap1 in the cytoplasm. Nrf-2 is dissociated from the Nrf-2/Keap1 complex and translocated to the nucleus by stimuli such as UVB irradiation. As a result, heme oxygenase-1 (HO-1) and antioxidant enzymes superoxide dismutase (SOD), glutathione peroxidase (GPx), and catalase (CAT) are upregulated. Thus, when the signaling pathway of Nrf-2/HO-1 is stimulated, the expression of antioxidant related factors is activated to maintain the oxidation-reduction homeostasis of cells [13]. Therefore, restoring various signaling pathways related to UV irradiation could be an effective strategy in preventing photoaging.

The Pacific whiting (*Merluccius productus*) is a fish found in the west coast of the United States and Canada. Annual yields have been recorded in the range of 100,000 to 350,000 tonnes, and higher harvests are now expected in the USA [14]. As the yield of fish increases, fish’s skins, bones, and intestines are thrown away as by-products of processing. Among them, fish skin continues to be the material of interest for skin health as a good source of gelatin [15]. Although it has been reported that the protein hydrolysate of Pacific whiting showed good antioxidant activity, further research on Pacific whiting is still needed, especially focusing on its byproduct, to better utilize this abundant yet underutilized resource [16]. In the current study, we investigated the anti-photoaging effect of Pacific whiting skin gelatin (PWG) and investigated the molecular mechanism involved to assess the potential value of PWG in skin health application.

## 2. Results

### 2.1. Cell Viability of PWG

**Figure 1:**
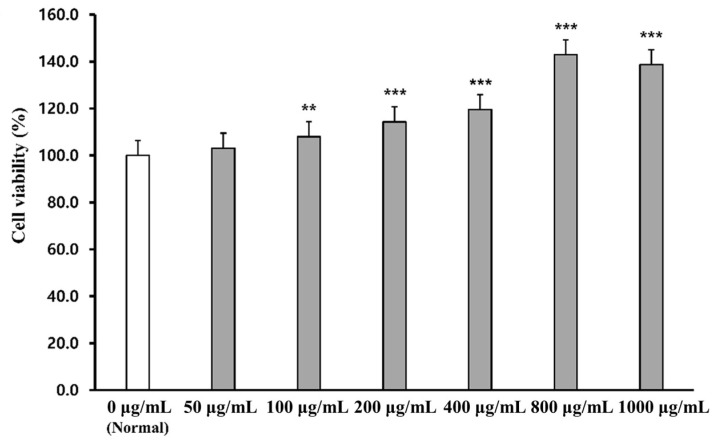
Cell viability in PWG-treated HDFs. HDFs were treated with different concentrations of PWG and incubated for 24 h. After incubation, MTT assay was performed to measure cell viability of PWG. All data were indicated as mean ± SD of at least three independent experiments. ** *p* < 0.01 and *** *p* < 0.001 versus normal.

The effect of PWG on cell viability was assessed by MTT assay in human dermal fibroblasts (HDFs) (Figure 1). HDFs were treated with PWG at a range of concentrations (50, 100, 200, 400, 800, and 1000 μg/mL). The cell viability of PWG-treated HDF was not only non-cytotoxic up to 1000 μg/mL but also had a cell proliferation effect. Therefore, concentrations of PWG were set at 50, 100, and 200 μg/mL for subsequent experiments.

### 2.2. PWG Stimulates Type I Procollagen Synthesis and Inhibits MMP1 Synthesis in UVB-Irradiated HDFs

**Figure 2:**
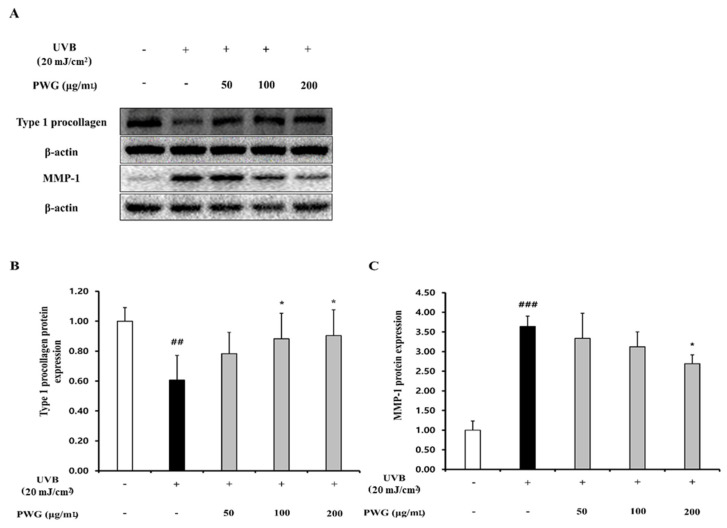
PWG stimulates type I procollagen synthesis and inhibits MMP1 synthesis in UVB-irradiated HDFs. HDFs were irradiated with UVB (20 mJ/cm^2^), followed by treatment with 50, 100, and 200 µg/mL of PWG. Protein expression of type I procollagen (**B**) and MMP-1 (**C**) were determined using western blot analysis (**A**). All data were indicated as mean ± SD of at least three independent experiments. ^##^ *p* < 0.01 and ^###^ *p* < 0.001 versus normal, * *p* < 0.05 versus UVB-irradiated cells.

We measured the protein expression of type I procollagen and MMP1 in HDFs, the main factors for the anti-wrinkle effect (Figure 2). The protein expression of type I procollagen in UVB-irradiated HDFs was reduced by 0.6-fold compared to the control group. However, in HDFs treated with PWG, compared with the UVB-irradiated group, type I procollagen expression recovered 45%, 70%, and 76% at 50, 100, and 200 μg/mL, respectively, in a dose-dependent manner. In contrast, MMP1 protein expression of UVB-irradiated HDFs was increased 3.6-fold compared to the control. However, after PWG treatment, MMP1 decreased by 12%, 26%, and 36% at 50, 100, and 200 μg/mL, respectively, in a dose-dependent manner compared with the UVB-irradiated group. These results indicate that PWG has a protective effect against photoaging by upregulating type I procollagen and downregulating MMP1 in UVB-irradiated HDFs.

### 2.3. PWG Stimulates TGF-β/Smad Signaling Pathway in UVB-Irradiated HDFs

**Figure 3:**
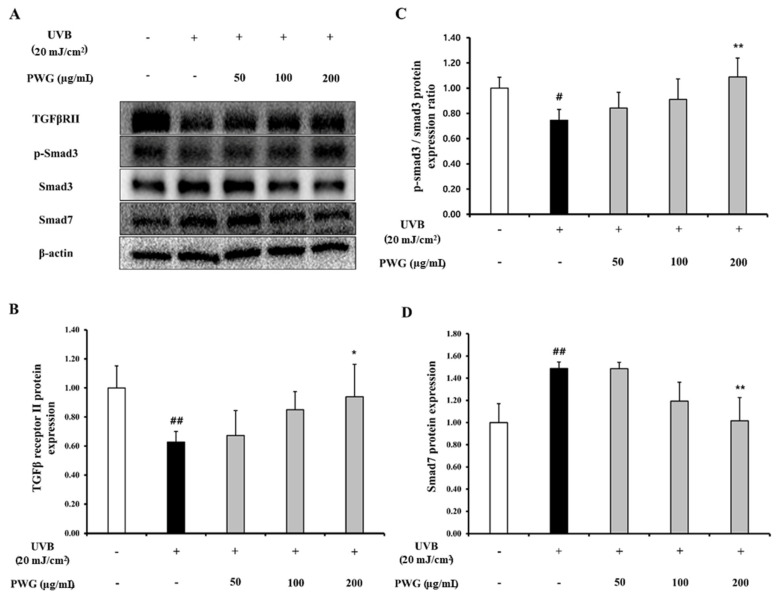
PWG stimulates TGF-β/Smad signaling pathway in UVB-irradiated HDFs. HDFs were irradiated with UVB (20 mJ/cm^2^), followed by treatment with 50, 100, and 200 µg/mL of PWG. Protein expression of TGFβRII (**B**), p-Smad3, Smad3 (**C**), and Smad7 (**D**) were evaluated by western blot (**A**). All data were indicated as mean ± SD of at least three independent experiments. ^#^ *p* < 0.05, ^##^ *p* < 0.01 versus normal, * *p* < 0.05, ** *p* < 0.01 versus UVB-irradiated cells.

We investigated the effects of PWG on TGF-β/Smad signaling pathway in UVB-irradiated HDFs (Figure 3). The protein expression of TGF-β receptor II in UVB-irradiated HDFs was diminished by 0.63-fold compared to the control. After PWG treatment, TGF-β receptor II recovered by 12%, 60%, and 84% at 50, 100, and 200 μg/mL, respectively, compared with the UVB-irradiated group. Also, phosphorylation of Smad3 in UVB-irradiated HDF cells was decreased by 0.75-fold compared to the control. However, after PWG treatment, compared with the UVB-irradiated group, Smad3 phosphorylation recovered 38%, and 65% at 50, 100 μg/mL, respectively, and normalized at 200 μg/mL. Also, the protein expression of Smad7 was increased 1.49-fold in UVB-irradiated HDFs compared to the control. PWG is dose-dependently reduced the protein expression of Smad7 by 1%, 60%, and 97% at 50, 100, and 200 μg/mL, respectively, compared to the UVB-induced group. These results suggest that PWG stimulates the TGF-β/Smad signaling pathway in UVB-irradiated skin cells.

### 2.4. PWG Inhibits MAPK Signaling Pathway in UVB-Irradiated HDFs

**Figure 4:**
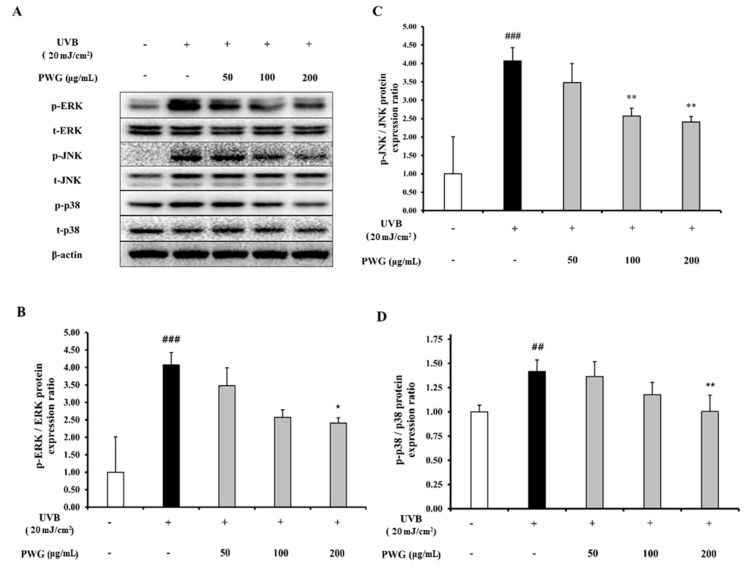
PWG inhibits MAPK signaling pathway in UVB-irradiated HDFs. HDFs were irradiated with UVB (20 mJ/cm^2^), followed by treatment with 50, 100, and 200 µg/mL of PWG. Phosphorylation and total protein ratios of ERK (**B**), JNK (**C**), and p38 (**D**) were determined using western blot analysis (**A**). All data were indicated as mean ± SD of at least three independent experiments. ^##^ *p* < 0.01 and ^###^ *p* < 0.001 versus normal, * *p* < 0.05 and ** *p* < 0.01 versus UVB-irradiated cells.

We determined the inhibitory effect of PWG on the phosphorylation of MAPKs in UVB-irradiation HDFs (Figure 4). In UVB-irradiated HDFs, phosphorylation of JNK, ERK, and p38 were increased 4.1, 2.1, and 1.4-fold compared to the control. However, compared to the UVB-irradiation group, PWG significantly reduced phosphorylation of MAPKs in a dose-dependent manner by 19%, 49%, 54% in JNK, 3%, 20%, 46% in ERK, and 12%, 57%, 99% in p38 at 50, 100, and 200 μg/mL, respectively. Among MAPKs, phosphorylation of p38 was normalized by PWG treatment. These results suggest that PWG inhibits the MAPK signaling pathway in UVB-irradiated HDFs.

### 2.5. PWG Inhibits AP-1 Expression in UVB-Irradiated HDFs

**Figure 5:**
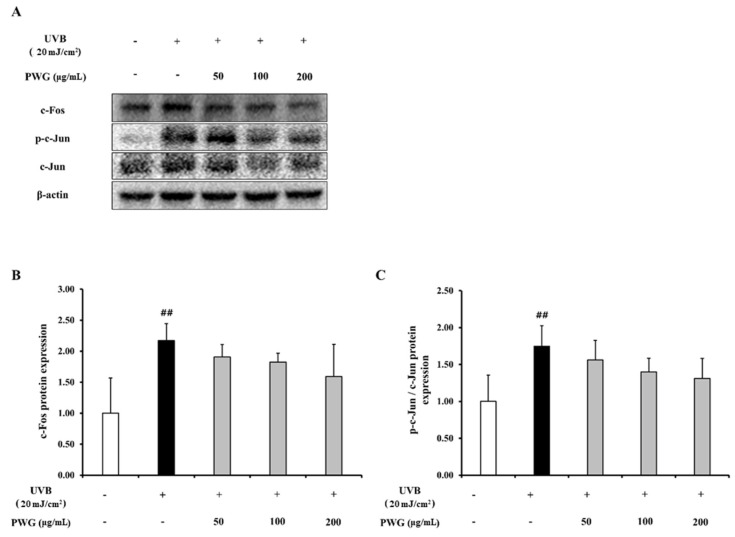
PWG inhibits AP-1 expression in UVB-irradiated HDFs. HDFs were irradiated with UVB (20 mJ/cm^2^), followed by treatment with 50, 100, and 200 µg/mL of PWG. Protein expressions of c-Fos (**B**), c-Jun, p-c-Jun (**C**) were determined by western blot (**A**). All data were indicated as mean ± SD of at least three independent experiments. ^##^ *p* < 0.01 versus normal.

In UVB-irradiated HDFs, the protein expression of c-Fos and phosphorylation of c-Jun, which are subunits of AP-1, increased 2.2-fold and 1.8-fold compared to the control (Figure 5). On the contrary, protein expression of c-Fos was diminished by 23%, 30%, and 50% at 50, 100, and 200 μg/mL of PWG, respectively, compared with the UVB-irradiated group. Also, PWG reduced phosphorylation of c-Jun by 25%, 46%, and 58% at 50, 100, and 200 μg/mL, respectively, compared with the UVB-irradiated group. While no statistical significance was found, the results suggested the trend that PWG downregulated the protein expression of c-Fos and the phosphorylation of c-Jun.

### 2.6. PWG Inhibits NF-κB Signaling Pathway in UVB-Irradiated HDFs

**Figure 6:**
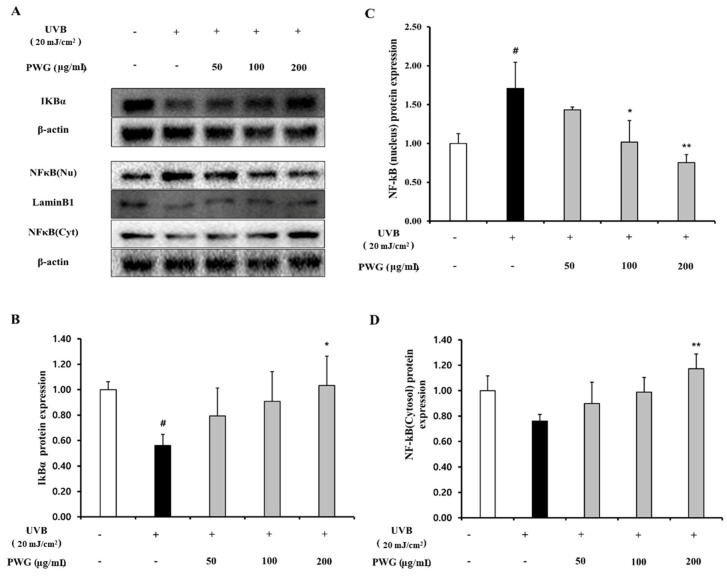
PWG inhibits NFκB signaling pathway in UVB-irradiated HDFs. HDFs were irradiated with UVB (20 mJ/cm^2^), followed by treatment with 50, 100, and 200 µg/mL of PWG. Protein expression of IKBα (**B**), and NFκB (**C**,**D**) in the nucleus and cytoplasm were determined by western blot (**A**). All data were indicated as mean ± SD of at least three independent experiments. ^#^ *p* < 0.05 versus normal, * *p* < 0.05, ** *p* < 0.01 versus UVB-irradiated cells.

**Figure 7:**
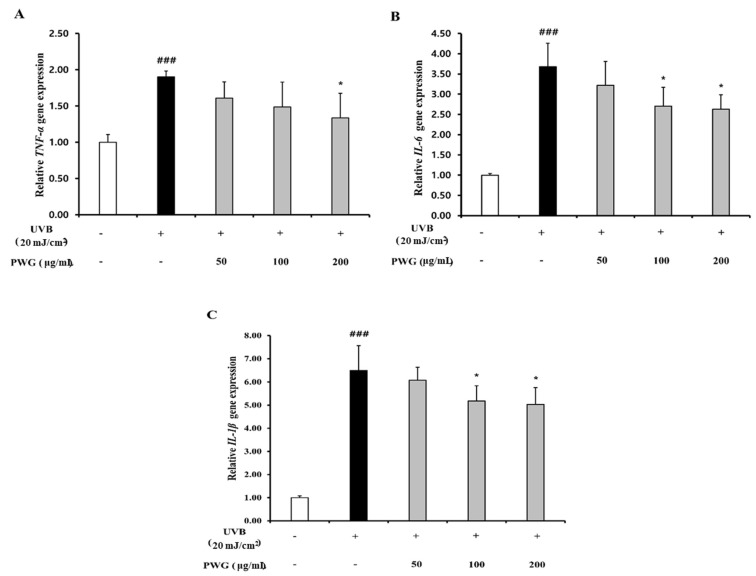
PWG inhibits inflammatory cytokines in UVB-irradiated HDFs. HDFs were irradiated with UVB (20 mJ/cm^2^), followed by treatment with 50, 100, and 200 µg/mL of PWG. Gene expression of TNF-α (**A**), IL-6 (**B**), and IL-1β (**C**) were determined by qRT-PCR. All data were indicated as mean ± SD of at least three independent experiments. ^###^ *p* < 0.001 versus normal, * *p* < 0.05, versus UVB-irradiated cells.

We examined the inhibitory effect of PWG on the NF-κB signaling pathway in UVB-irradiated HDFs (Figure 6). In the cytoplasm, IκBα protein expression of UVB-irradiated HDFs was reduced by 0.56-fold compared to the control. After PWG treatment, compared with the UVB-irradiated group, IκBα was recovered by 53% and 79% at 50 and 100 μg/mL, respectively, and normalized at 200 μg/mL. In the cytoplasm of UVB-irradiated HDFs, the expression of NF-κB protein was reduced by 0.76-fold compared to normal. But after PWG treatment, compared with the UVB-irradiated group, NF-κB was recovered by 58%, 95% at 50 and 100 μg/mL, respectively, and normalized at 200 μg/mL. Furthermore, NF-κB protein expression in the nucleus of UVB-irradiated HDFs was increased 1.71-fold compared to the control. After PWG treatment, compared with the UVB-irradiated group, NF-κB recovered it by 39%, 97% at 50, 100 μg/mL, respectively, and normalized at 200 μg/mL. In addition, we investigated the change in gene expression of pro-inflammatory cytokines, TNF-α, IL-1β, and IL-6 (Figure 7). The gene expression of TNF-α, IL-6, and IL-1β of UVB-irradiated HDFs was increased by 1.90, 3.68, and 6.50 folds, respectively, compared to the control. In contrast, PWG treatment reduced gene expression of TNF-α by 32%, 46%, and 63% at 50, 100, and 200 μg/mL, respectively, compared with the UVB-irradiated group. Also, IL-1β and IL-6 gene expressions were reduced by 8%, 24%, 27% and 17%, 36%, and 39% at PWG 50, 100 and 200 μg/mL, respectively. These results suggest that PWG downregulates the gene expression of pro-inflammatory cytokines through inhibition of NF-κB translocation.

### 2.7. PWG Activates Nrf-2/HO-1 Signaling Pathway in UVB-Irradiated HDFs

**Figure 8:**
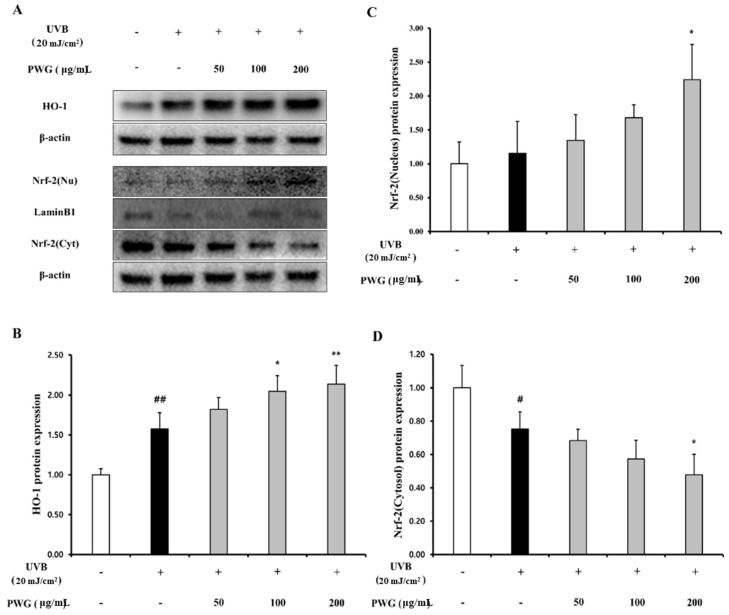
PWG activates Nrf-2/HO-1 signaling pathway in UVB-irradiated HDFs. HDFs were irradiated with UVB (20 mJ/cm^2^), followed by treatment with 50, 100, and 200 µg/mL of PWG. HO-1 (**B**) and Nrf-2 (**C**), (**D**) protein expression in the cytoplasm and nucleus was performed by western blot (**A**). All data were indicated as mean ± SD of at least three independent experiments. ^#^ *p* < 0.05, ^##^ *p* < 0.01, versus normal, * *p* < 0.05, ** *p* < 0.01 versus UVB-irradiated cells.

**Figure 9:**
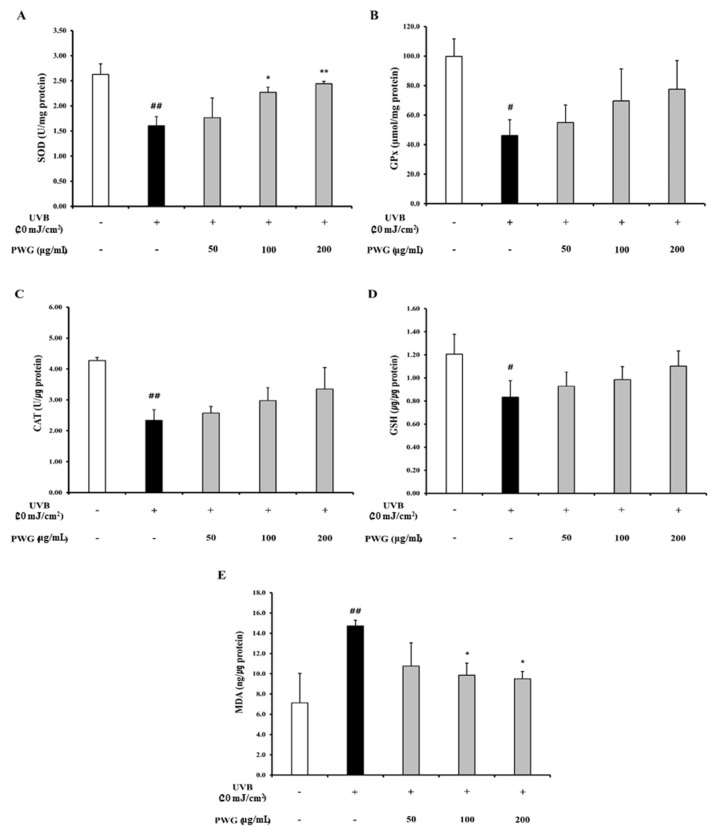
Effects of PWG on the activity of antioxidant enzymes in UVB-irradiated HDFs. HDFs were irradiated with UVB (20 mJ/cm^2^), followed by treatment with 50, 100, and 200 µg/mL of PWG. Activity of SOD (**A**), GPx (**B**), CAT (**C**), content of GSH (**D**), and MDA (**E**) were detected in HDF cells using ELISA kits. All data were indicated as mean ± SD of at least three independent experiments. ^#^ *p* < 0.05, ^##^ *p* < 0.01, versus normal, * *p* < 0.05, ** *p* < 0.01 versus UVB-irradiated cells.

We investigated the effects of PWG on the Nrf-2/HO-1 signaling pathway in UVB-irradiated HDFs (Figure 8). In the cytoplasm, the Nrf-2 in UVB-irradiated HDFs was decreased by 0.75-fold compared to the control. After PWG treatment, Nrf-2 was decreased by 9%, 24%, and 37% at 50, 100, and 200 μg/mL, respectively, compared with the UVB-irradiated group. On the other hand, the nucleus translocation of Nrf-2 in the UVB-irradiated HDFs was increased by 1.15-fold compared to the control. However, after PWG treatment, the nucleus translocation of Nrf-2 was increased by 17%, 46%, and 94% at 50, 100, and 200 μg/mL, respectively, compared with the UVB-irradiation group. Also, we evaluated the HO-1 protein expression, an antioxidant factor activated by Nrf-2, in the cytoplasm of UVB-irradiated HDFs. The HO-1 protein expression in UVB-irradiated HDFs was increased by 1.58-fold compared to the control. After PWG treatment, the protein expression of HO-1 was increased by 15%, 30%, and 36% at 50, 100, and 200 μg/mL, respectively, compared with the UVB-induced group. We also estimated SOD, CAT, GPx activity and GSH content, MDA production in HDFs (Figure 9). In UVB-irradiated HDFs, SOD, CAT, GPx activity, and GSH content were decreased while MDA production was increased. However, PWG significantly increased SOD activity and increased CAT, GPx, activities and GSH content in a dose-dependent manner without statistical significance. In addition, MDA production was significantly reduced. These results suggest that PWG is protective against skin photoaging by upregulating antioxidant enzymes via activation of the Nrf-2/HO-1 signaling pathway.

## 3. Discussion

In this study, we found that PWG was effective in anti-photoaging by stimulating type I procollagen protein expression and decreasing MMP1 protein expression in UVB-damaged HDFs. UVB-irradiation is known to activate the MAPK/AP-1 and NF-κB signaling pathways and impair the TGF-β/Smad signaling pathway, which was a mechanism associated with photoaging [17,18,19]. Furthermore, the Nrf-2/HO-1 signaling pathway attenuates damage to oxidative stress [20]. We investigated whether PWG affects 4 types of molecular mechanisms, which were collagen synthesis (TGF-β/Smad pathway), collagen degradation (MAPK/AP-1 pathway), inflammation involved in photodamage (NF-κB pathway), and antioxidant effects (Nrf-2/HO-1 pathway).

TGF-β/Smad is a representative signaling pathway involved in collagen synthesis. The TGF-β receptors are divided into two types: TβRI and TβRII, and when a ligand binds to the receptor, TβRI and TβRII bind to form a dimer. And then, TβRII phosphorylates TβRI, and the activated TβRI recognizes and phosphorylates Smad3, one of the ligand-specific receptor-activated Smad proteins (R-Smad). As a result, phosphorylated Smad3 forms a complex with common partner Smads (co-Smad) and then translocated to the nucleus, contributing to procollagen synthesis. UVB irradiation decreases TβRII to downregulate the Smad3 phosphorylation and reduces collagen synthesis [18,21,22]. It also upregulates Smad7, which antagonizes Smad3 phosphorylation by binding to TβRI [10,23]. Accordingly, we found that PWG restored expression of TβRII downregulated by UVB and stimulated phosphorylation of Smad3. Also, PWG downregulated Smad7 protein expression, thus helping Smad3 phosphorylation. These results demonstrate that PWG promoted collagen synthesis in photoaging skin by activating the TGF-β/Smad signaling pathway and suppressing the expression of Smad7.

The GTP binding proteins Rac, Ras, and Cdc42 are activated on the fibroblast cell surface when the skin is exposed to UV. Ras contributes to ERK activation by recruiting Raf-1 from the plasma membrane. Rac and Cdc42 bind to MEKK1 and activate MAPK, which was composed ERK, JNK, and p38 [18]. Phosphorylated ERK plays an important role in the trigger for c-Fos induction [24]. Also, phosphorylated JNK and p38 promote phosphorylation of c-Jun [25]. Phosphorylated c-Jun combines with c-Fos to form a heterodimer, AP-1, which binds to target gene promoters. Finally, AP-1 promotes the expression of MMP1, which is responsible for the degradation of fibrillar type 1 collagen. We confirmed that PWG significantly suppressed the phosphorylation of JNK, ERK, and p38 overall. Especially, PWG normalized phosphorylation of p38 among MAPKs. PWG inhibited ERK phosphorylation to decrease c-Fos expression, and inhibited p38 and JNK phosphorylation to decrease c-Jun phosphorylation. These reductions of c-Fos and phosphorylated c-Jun, while not significant, suggested that the formation of AP-1 was reduced, thereby suppressing the expression of MMP-1. The activation of p38 by UVB was reported to stimulate NF-κB and induce an inflammatory response [26,27]. Our results suggest that PWG can suppress the inflammatory response by inhibiting the NF-κB pathway via normalization of the phosphorylation of p38.

NF-κB is induced by stimuli such as UV irradiation and oxidative stress and is known as a mediator of inflammatory responses and apoptosis. Also, NF-κB is another pivotal regulator for the transcriptional activity of MMP1 in response to UVB irradiation [28]. In the normal state, NF-κB is controlled by combining with IκBα, which inhibits NF-κB translocation into the nucleus. However, when exposed to UV, IκBα is phosphorylated and degraded by the proteasomal ubiquitination process. As a result, NF-κB is translocated to the nucleus and upregulates MMP1 [29]. In our study, PWG normalized the expression of IκBα and also normalized the expression of NF-κB in the cytoplasm and nucleus. These results demonstrated that PWG restores the expression of IκBα damaged by UVB and suppresses the translocation of NF-κB to the nucleus. Our result suggests that PWG may suppress MMP1 expression via the NF-κB signaling pathway as one of the main target pathways. NF-κB translocation into the nucleus increases pro-inflammatory cytokines such as TNF-α, IL-6, and IL-1β involved in photodamage. TNF-α preferentially activates and accelerates MAPK/AP-1 to increase collagen degradation [30]. IL-1β damages the TGF-β signaling pathway involved in collagen synthesis [31], and IL-6 upregulates the expression of MMPs [32]. In the UVB-irradiated group, gene expressions of pro-inflammatory cytokines were significantly increased. However, we confirmed that PWG significantly reduced the gene expression of TNF-α, IL-6, and IL-1β in UVB-irradiation HDFs. These results suggest that PWG not only has the effect of suppressing the inflammatory response by reducing the expression of cytokines but also that it might be associated with collagen degradation and restoration of the TGF-β signaling pathway.

Nrf-2/HO-1 is a representative signaling pathway that protects the skin from oxidative stress. Nrf-2 binds to Keap1 in the cytoplasm in the normal state, but ROS production by UV irradiation dissociates Keap1, and Nrf-2 is translocated into the nucleus [33]. After translocated to the nucleus, Nrf-2 forms a heterodimer with Maf protein and binds to the ARE present in the promoter region of the antioxidant enzymes. Then, it increases the expression of HO-1 and antioxidant enzymes [12,34]. We confirmed that the Nrf-2 expression was increased in UVB-irradiated HDFs, followed by upregulation of HO-1 in the cytoplasm. We found that the upregulation of Nrf-2 was further enhanced by PWG treatment, and HO-1 was also increased. In addition, UVB irradiation reduces the activity of representative antioxidant enzymes SOD, CAT, GPx, which leads to decreased GSH content and accumulation of lipid peroxides, MDA [35,36]. PWG treatment increased antioxidant enzymes, restored the content of GSH, and decreased MDA production. A previous study has been reported that Nrf-2 overexpression of skin-derived precursors restored the activity of the antioxidant enzymes SOD, CAT, GPx, and GSH, which were reduced by UV irradiation [37]. These results suggest that Nrf-2 is increased by a compensatory response, and PWG further increases Nrf-2, thereby increasing the synthesis of antioxidant enzymes.

Our study has shown that PWG not only restores imbalances in collagen synthesis and degradation but also has anti-inflammatory and antioxidant properties. Taken together, our finding suggests PWG as a promising material for preventing skin photoaging. Analysis of the detailed chemical composition of PWG is currently underway to identify the compounds responsible for elicited bioactivity of PWG. Identification and characterization of the active component of PWG based on the current work will help enable the skin health application of PWG.

## 4. Materials and Methods

### 4.1. Materials

Human dermal fibroblasts (HDFs) were purchased from American Type Culture Collection (ATCC No.PCS-201-012^™^, Manassas, VA, USA). Glutathione (GSH) ELISA kit (catalog#: E-EL-0026, Bethesda, MD, USA), malondialdehyde (MDA) ELISA kit (catalog#: E-EL-0060), and glutathione peroxidase (GPx) assay kit were purchased from Elabscience (catalog #: BC0096). Superoxide dismutase (SOD) assay kit was obtained from Cayman Chemical Co (catalog #: 706,002, Ann Arbor, MI), and catalase (CAT) assay kit was purchased from Biomax (catalog#: BO-CAT-400, Seoul, Korea). Antibodies against Type I procollagen and β-actin were purchased from Santa Cruz Biotechnology (Santa Cruz, CA, USA). Antibodies against Smad-7 were purchased from R&D Systems (Minneapolis, MN, USA). Antibodies against Smad-3, phosphorylated-Smad-3 (p-smad-3), TGFβ receptor II, JNK, phosphorylated-JNK (p-JNK), ERK, phosphorylated-ERK (p-ERK), p38, phosphorylated-p38 (p-p38), c-Fos, phosphorylated-c-Jun (p-c-Jun), c-Jun, MMP1, NF-κB p65, IκB alpha, and Lamin B1 were purchased from Cell Signaling Technology (Danvers, MA, USA). All the materials and chemicals used in this study were of the highest analytical grade.

### 4.2. Preparation of Pacific Whiting Skin Gelatin (PWG)

Pacific whiting was obtained from Pacific Seafood (Clackamas, OR, USA) and skin was separated on ice. Gelatin extraction from Pacific whiting skin was carried out in the manner described by Fan et al., with minor adjustments [38]. First, for every 1 g of frozen skin, 4 mL of ice-cold 0.45 M NaCl was used to rinse the skin for 3 min. Next, the skin was washed twice with deionized (DI) H_2_O. The skin was then incubated in 4 mL of 50 mM tris-HCl (pH 8) for every 1 g of skin and 1.5 U/g trypsin for 4 h at 37 °C. After incubation, the skin was again washed twice with DI H_2_O. Next, 4 mL of fresh DI H_2_O was added to each 1 g of skin, and the mixture was gently stirred at 45 °C for 6 h. After stirring, the mixture was centrifuged at 7000× *g*, and 15 °C for 30 min. Immediately following centrifugation, the gelatin containing supernatant was lyophilized, and stored at −20 °C.

### 4.3. Cell Culture

Human dermal fibroblasts (HDFs) were purchased from American Type Culture Collection (ATCC No.PCS-201-012^™^, Manassas, VA, USA) and cultured in Dulbecco’s modified Eagle’s medium (DMEM, Welgene, Korea) supplemented with 10% fetal bovine serum (FBS) (Hyclone, UT, USA) and 1% penicillin-streptomycin (Welgene, Korea). The cells were grown at 37 °C, 5% CO_2_ incubator. Culture media were changed every three days, and the cells were subcultured when the cells were grown to 80% confluence. All experiments used between 6 and 10 passages with HDFs.

### 4.4. UVB Irradiation and PWG Treatment

The UVB dose was determined as 20 mJ/cm^2^ based on the cell viability of HDFs. Before UVB irradiation, HDF was washed twice with phosphate-buffered saline (PBS) and replaced instead of medium. HDFs replaced with PBS were exposed to UVB for 20 s to reach UVB (20 mJ/cm^2^) (306 nm, G8T5E, 8 W, Sankyo Denki, Japan). After UVB irradiation, cells were changed to a serum-free medium containing different concentrations of PWG and were incubated for 24 h. All of the cell experiments were performed three times.

### 4.5. Cell Viability Assay

Cell viability of PWG was measured by MTT assay. MTT reagent was purchased from Sigma-Aldrich (St. Louis, MO, USA). HDFs were seeded at 1 × 10^4^ per well in 96 well plate and incubated at 37 °C, 5% CO_2_. The cells were treated with serum-free media containing PWG for each concentration and then incubated for 24 h. After incubation, the media was replaced with MTT reagent (0.5 mg/mL), and the cells were incubated for 3 h. Then, the reagent was suctioned, and formazan crystals were dissolved in dimethyl sulfoxide (DMSO, Duksan, Korea). The Cell viability was measured at 540 nm absorbance in an ELISA microplate reader (Bio-Tek Instruments Inc., Winooski, VT, USA).

### 4.6. Determination of Antioxidant Enzyme Activity

HDFs were cultured in 6-wells and then exposed to UVB (20mJ/cm^2^). The exposed HDFs were treated with PWG at different concentrations for 24 h. Glutathione (GSH) ELISA kit from Elabscience (catalog#: E-EL-0026, Bethesda, MD, USA), malondialdehyde (MDA) ELISA kit from Elabscience (catalog#: E-EL-0060) superoxide dismutase (SOD) assay kit from Cayman Chemical Co (catalog #: 706,002, Ann Arbor, MI, USA), glutathione peroxidase (GPx) assay kit from Elabscience (catalog #: BC0096), and catalase (CAT) assay kit (Biomax, Seoul, Korea) were measured in cell lysates. All assay kits were performed according to the manufacturer’s protocol.

### 4.7. RNA Extraction and Quantitative Real-Time Polymerase Chain Reaction (qRT-PCR) Analysis

The total RNA of HDFs was extracted according to the protocol of the Easy-RED™ total RNA extraction kit (iNtRON Biotechnology, Gyeonggi-do, Korea). After RNA extraction, cDNA was synthesized using a cDNA synthesis kit (Takara, Shiga, Japan). And then, qRT-PCR analysis was performed with SYBR Premix Ex Taq (Takara, Shiga, Japan) using an ABI StepOnePlus™ Real-Time PCR machine (Applied Biosystems, Foster, CA, USA). The primer sequences are shown in Table 1. All results were normalized to glyceraldehyde 3-phosphated dehydrogenase (GAPDH).

### 4.8. Western Blot Analysis

The HDFs were lysed in RIPA II Cell Lysis Buffer(1X) (GenDEPOT, USA) containing protease inhibitor cocktails (Roche, Mannheim, Germany) and PhosSTOP EASYpack phosphatase inhibitor cocktail tablets (Roche, Mannheim, Germany). HDFs lysates were dispersed ultrasonically for 20 min and centrifuged at 13,000 rpm for 15 min at 4 °C. Nuclear and cytosol fractionations were conducted according to the manufacturer’s instructions of the Nuclear Extraction Kit (Abcam, Cambridge, MA, USA). Protein concentration was adjusted using PierceTM BCA Protein Assay Kit (Thermo Fisher Scientific, Waltham, MA, USA), and the same amount of protein was separated from SDS-PAGE gel. Then, proteins were transferred to a polyvinylidene fluoride (PVDF) membrane. Thereafter, the membranes were blocked with a blocking solution containing 5% skim milk in Tris-buffered saline containing Tween 20 (TBST) for 1 h and incubated overnight with primary antibody diluted in 5% BSA in TBST at 4 °C. And then, the membrane was rinsed with TBST in 30 min and incubated with horseradish peroxidase-conjugated secondary antibodies in blocking solution (Santa Cruz Biotechnology Inc., Santa Cruz, CA, USA) for 2 h. All bands were visualized by using ChemiDocTM XRS+System (Bio-Rad, Richmond, CA, USA). The primary antibodies are as follows: Type I procollagen, β-actin (Santa Cruz Biotechnology Inc., Santa Cruz, CA, USA), Smad-7 (R&D systems Inc., Minneapolis, USA), Smad-3, phosphorylated-Smad-3 (p-smad-3), TGFβ receptor II, JNK, phosphorylated-JNK (p-JNK), ERK, phosphorylated-ERK (p-ERK), p38, phosphorylated-p38 (p-p38), c-Fos, phosphorylated-c-Jun (p-c-Jun), c-Jun, MMP1, NF-κB p65, IκB alpha, and Lamin B1 (Cell Signaling Technology, Danvers, MA, USA).

### 4.9. Statistical Analysis

All results were performed in at least three independent experiments. Results were conducted to statistical analysis using Statistical Packages for Social Science (SPSS) 25 software (SPSS Inc., Chicago, IL, USA) and analyzed by one-way analysis of variance (ANOVA) followed by Tukey’s post hoc test. The data were shown as the mean ± standard deviation (SD). Statistical significance was set at ^#^ *p* < 0.05, ^##^ *p* < 0.01, and ^###^ *p* < 0.001 compared to the untreated cells; * *p* < 0.05, ** *p* < 0.01, and *** *p* < 0.001 compared to the UVB-irradiated cells.

## 5. Conclusions

Our data show that PWG inhibits skin photoaging by recovering type I procollagen and decreasing MMP1. PWG activated collagen synthesis (TGF-β/Smad pathway) and inhibited collagen degradation (MAPK/AP-1 pathway), recovering the imbalance of collagen caused by UVB-irradiation. In addition, PWG suppressed the inflammatory response (NF-κB pathway) caused by UVB-irradiation and reduced the pro-inflammatory cytokines gene expression. Lastly, PWG increased antioxidant enzyme activity (Nrf-2/HO-1 pathway) and protected the skin from oxidative stress. Taken together, the current study indicates PWG may be used as a potential ingredient for anti-photoaging effects.

## Figures and Tables

**Table 1:** The oligonucleotide primer sequences used in RT-qPCR.

Gene	Sequence
*Gapdh*	F: 5′- TCGACAGTCAGCCGCATCTTCTTT -3′ R: 5′- ACCAAATCCGTTGACTCCGACCTT -3′
*IL-6*	F: 5′- CACAGACAGCCACTCACCTC -3′ R: 5′- TTTTCTGCCAGTGCCTCTTT -3′
*IL1-β*	F: 5′- GGACAAGCTGAGGAAGATGC -3′ R: 5′- TCGTTATCCCATGTGTGTCGAA -3′
*TNF-α*	F: 5′- CAAAGTAGACCTGCCCAGAC -3′ R: 5′- GACCTCTCTCTAATCAGCCC -3′

## Data Availability

Not applicable.

## References

[1] Hwang K.-A., Bo-Rim Y., Kyung-Chul C. (2011). Molecular Mechanisms and in Vivo Mouse Models of Skin Aging Associated with Dermal Matrix Alterations. Lab. Anim. Res..

[2] Naylor C.E., Watson R.E.B., Sherratt M.J. (2011). Molecular Aspects of Skin Ageing. Maturitas.

[3] Matsumura Y., Ananthaswamy H.N. (2004). Toxic Effects of Ultraviolet Radiation on the Skin. Toxicol. Appl. Pharmacol..

[4] Scharffetter–Kochanek K., Brenneisen P., Wenk J., Herrmann G., Ma W., Kuhr L., Meewes C., Wlaschek M. (2000). Photoaging of the Skin from Phenotype to Mechanisms. Exp. Gerontol..

[5] Ganceviciene R., Liakou A.I., Theodoridis A., Makrantonaki E., Zouboulis C.C. (2012). Skin Anti-Aging Strategies. Dermato-endocrinology.

[6] Verma R.P., Hansch C. (2007). Matrix Metalloproteinases (Mmps): Chemical–Biological Functions and (Q) Sars. Bioorg. Med. Chem..

[7] Yamamoto Y., Gaynor R.B. (2004). Ikappab Kinases: Key Regulators of the Nf-Kappab Pathway. Trends Biochem. Sci..

[8] Quan T., Shao Y., He T., Voorhees J.J., Fisher G.J. (2010). Reduced Expression of Connective Tissue Growth Factor (Ctgf/Ccn2) Mediates Collagen Loss in Chronologically Aged Human Skin. J. Investig. Dermatol..

[9] Varga J., Rosenbloom J., Jimenez S.A. (1987). Transforming Growth Factor Β (Tgf Β) Causes a Persistent Increase in Steady-State Amounts of Type I and Type Iii Collagen and Fibronectin Mrnas in Normal Human Dermal Fibroblasts. Biochem. J..

[10] He T., Quan T., Shao Y., Voorhees J.J., Fisher G.J. (2014). Oxidative Exposure Impairs Tgf-Β Pathway Via Reduction of Type Ii Receptor and Smad3 in Human Skin Fibroblasts. Age.

[11] Brand R.M., Wipf P., Durham A., Epperly M.W., Greenberger J.S., Falo L.D.J. (2018). Targeting Mitochondrial Oxidative Stress to Mitigate Uv-Induced Skin Damage. Front. Pharmacol..

[12] Itoh K., Chiba T., Takahashi S., Ishii T., Igarashi K., Katoh Y., Oyake T., Hayashi N., Satoh K., Hatayama I. (1997). An Nrf2/Small Maf Heterodimer Mediates the Induction of Phase Ii Detoxifying Enzyme Genes through Antioxidant Response Elements. Biochem. Biophys. Res. Commun..

[13] Zhang D.D. (2006). Mechanistic Studies of the Nrf2-Keap1 Signaling Pathway. Drug Metab. Rev..

[14] Mann H.M. (2008). Achievements of the Pacific Whiting Conservation Cooperative: Rational Collaboration in a Sea of Irrational Competition. Case Stud. Fish. Self-Gov..

[15] Boran G., Regenstein J.M. (2010). Fish Gelatin. Adv. Food Nutr. Res..

[16] Cheung I.W.Y., Cheung L.K.Y., Tan N.Y., Li-Chan E.C.Y. (2012). The Role of Molecular Size in Antioxidant Activity of Peptide Fractions from Pacific Hake (Merluccius Productus) Hydrolysates. Food Chem..

[17] Yaar M., Gilchrest B.A. (2007). Photoageing: Mechanism, Prevention and Therapy. Br. J. Dermatol..

[18] Rittié L., Fisher G.J. (2002). Uv-Light-Induced Signal Cascades and Skin Aging. Ageing Res. Rev..

[19] Kim Y.J., Lee H.E., Cho E.-B., Kim D.-H., Kim B.O., Kang I.K., Jung H.Y., Cho Y.J. (2019). Protective Effects of Galangin against Uvb Irradiation-Induced Photo-Aging in Ccd-986sk Human Skin Fibroblasts. Appl. Biol. Chem..

[20] Gao W., Wang Y.S., Hwang E., Lin P., Bae J., Seo S.A., Yan Z., Yi T.H. (2018). *Rubus Idaeus* L. (Red Raspberry) Blocks Uvb-Induced Mmp Production and Promotes Type I Procollagen Synthesis Via Inhibition of Mapk/Ap-1, Nf-Κβ and Stimulation of Tgf-Β/Smad, Nrf2 in Normal Human Dermal Fibroblasts. J. Photochem. Photobiol. B Biol..

[21] Cavinato M., Jansen-Dürr P. (2017). Molecular Mechanisms of Uvb-Induced Senescence of Dermal Fibroblasts and Its Relevance for Photoaging of the Human Skin. Exp. Gerontol..

[22] Verrecchia F., Mauviel A. (2002). Transforming Growth Factor-Β Signaling through the Smad Pathway: Role in Extracellular Matrix Gene Expression and Regulation. J. Investig. Dermatol..

[23] Chiang H.M., Chen C.W., Lin T.-Y., Kuo Y.-H. (2014). N-Phenethyl Caffeamide and Photodamage: Protecting Skin by Inhibiting Type I Procollagen Degradation and Stimulating Collagen Synthesis. Food Chem. Toxicol..

[24] Raman M., Chen W., Cobb M.H. (2007). Differential Regulation and Properties of Mapks. Oncogene.

[25] Fisher G.J., Talwar H.S., Lin J., Lin P., McPhillips F., Wang Z.Q., Li X., Wan Y., Kang S., Voorhees J.J. (1998). Retinoic Acid Inhibits Induction of C-Jun Protein by Ultraviolet Radiation That Occurs Subsequent to Activation of Mitogen-Activated Protein Kinase Pathways in Human Skin in Vivo. J. Clin. Investig..

[26] Kim A.L., Labasi J.M., Zhu Y., Tang X., McClure K., Gabel C.A., Athar M., Bickers D.R. (2005). Role of P38 Mapk in Uvb-Induced Inflammatory Responses in the Skin of Skh-1 Hairless Mice. J. Investig. Dermatol..

[27] Rahman M.M., Kundu J.K., Shin J.W., Na H.K., Surh Y.J. (2011). Docosahexaenoic Acid Inhibits Uvb-Induced Activation of Nf-Κb and Expression of Cox-2 and Nox-4 in Hr-1 Hairless Mouse Skin by Blocking Msk1 Signaling. Plos ONE.

[28] Muthusamy V., Piva T.J. (2010). The Uv Response of the Skin: A Review of the Mapk, Nfκb and Tnfα Signal Transduction Pathways. Arch. Dermatol. Res..

[29] Lee Y.-R., Noh E.M., Jeong E.Y., Yun S.K., Jeong Y.J., Kim J.-H., Kwon K.B., Kim B.S., Lee S.H., Park C.S. (2009). Cordycepin Inhibits Uvb-Induced Matrix Metalloproteinase Expression by Suppressing the Nf-Κb Pathway in Human Dermal Fibroblasts. Exp. Mol. Med..

[30] Kondo S. (2000). The Roles of Cytokines in Photoaging. J. Dermatol. Sci..

[31] Baugé C., Girard N., Leclercq S., Galéra P., Boumédiene K. (2012). Regulatory Mechanism of Transforming Growth Factor Beta Receptor Type Ii Degradation by Interleukin-1 in Primary Chondrocytes. Biochim. Biophys. Acta (BBA)-Mol. Cell Res..

[32] Ichihashi M., Ando H., Yoshida M., Niki Y., Matsui M. (2009). Photoaging of the Skin. Anti-Aging Med..

[33] Solomon H. (2019). The Nrf2/Ho-1 Axis as Targets for Flavanones: Neuroprotection by Pinocembrin, Naringenin, and Eriodictyol. Oxidative Med. Cell. Longev..

[34] Gęgotek A., Skrzydlewska E. (2015). The Role of Transcription Factor Nrf2 in Skin Cells Metabolism. Arch. Dermatol. Res..

[35] Ramachandran S., Prasad N.R., Karthikeyan S. (2010). Sesamol Inhibits Uvb-Induced Ros Generation and Subsequent Oxidative Damage in Cultured Human Skin Dermal Fibroblasts. Arch. Dermatol. Res..

[36] Wang C.-B., Ding B.X., Guo S.B., Wang Y.Z., Han Y.T., Wang Y.J. (2003). Protective Effect of Polypeptide from Chlamys Farreri on Mitochondria in Human Dermal Fibroblasts Irradiated by Ultraviolet B. Acta Pharmacol. Sin..

[37] Xian D., Xiong X., Xu J., Xian L., Lei Q., Song J., Zhong J. (2019). Nrf2 Overexpression for the Protective Effect of Skin-Derived Precursors against Uv-Induced Damage: Evidence from a Three-Dimensional Skin Model. Oxidative Med. Cell. Longev..

[38] Fan H., Dumont M.J., Simpson B.K. (2017). Extraction of Gelatin from Salmon (Salmo Salar) Fish Skin Using Trypsin-Aided Process: Optimization by Plackett–Burman and Response Surface Methodological Approaches. J. Food Sci. Technol..

